# Maximum entropy models provide functional connectivity estimates in neural networks

**DOI:** 10.1038/s41598-022-13674-4

**Published:** 2022-06-10

**Authors:** Martina Lamberti, Michael Hess, Inês Dias, Michel van Putten, Joost le Feber, Sarah Marzen

**Affiliations:** 1 Department of Clinical Neurophysiology, University of Twente, P.O. Box 217, 7500 AE Enschede, The Netherlands; 2 Laney Graduate School, Emory University, Atlanta, GA 30307 USA; 3 W. M. Keck Science Department, Pitzer, Scripps and Claremont McKenna College, Claremont, 91711 USA; 4 Clinical Neurophysiology TechMed Building, TL 3382, University of Twente, P.O. Box 217, 7500 AE Enschede, Netherlands

**Keywords:** Network models, Statistical methods

## Abstract

Tools to estimate brain connectivity offer the potential to enhance our understanding of brain functioning. The behavior of neuronal networks, including functional connectivity and induced connectivity changes by external stimuli, can be studied using models of cultured neurons. Cultured neurons tend to be active in groups, and pairs of neurons are said to be functionally connected when their firing patterns show significant synchronicity. Methods to infer functional connections are often based on pair-wise cross-correlation between activity patterns of (small groups of) neurons. However, these methods are not very sensitive to detect inhibitory connections, and they were not designed for use during stimulation. Maximum Entropy (MaxEnt) models may provide a conceptually different method to infer functional connectivity. They have the potential benefit to estimate functional connectivity during stimulation, and to infer excitatory as well as inhibitory connections. MaxEnt models do not involve pairwise comparison, but aim to capture probability distributions of sets of neurons that are synchronously active in discrete time bins. We used electrophysiological recordings from in vitro neuronal cultures on micro electrode arrays to investigate the ability of MaxEnt models to infer functional connectivity. Connectivity estimates provided by MaxEnt models correlated well with those obtained by conditional firing probabilities (CFP), an established cross-correlation based method. In addition, stimulus-induced connectivity changes were detected by MaxEnt models, and were of the same magnitude as those detected by CFP. Thus, MaxEnt models provide a potentially powerful new tool to study functional connectivity in neuronal networks.

## Introduction

Neuronal connectivity is essential for cognitive functions like learning and memory^1–3^, but is difficult to assess in the in vivo brain. Reduced models of networks of cultured neurons on micro electrode arrays have been used to investigate the relationship between connectivity and processes like memory formation^4,5^. Such networks present a wide range of responses to external stimuli^6,7^, and can produce diverse patterns from synchronous firing^8,9^ to chaotic trajectories^10^. Micro electrode arrays provide a useful tool to study cultured neural networks by recording action potentials from many neurons in parallel.

Network connectivity is often characterized by functional connectivity, which quantifies the likeliness of (groups of) neurons to fire in synchrony. In the last decades, various methods have been developed to infer functional connectivity, usually based on or related to cross correlation between activity patterns^11–14^. One such method is based on conditional firing probabilities (CFP), the likelihood of one neuron to fire, in response to another neuron firing^15^. This method uses spontaneous activity patterns to infer functional network connectivity, and yields strengths and latencies of excitatory connections between all pairs of active neurons. The finding that functional connections as obtained by CFP follow the rules of spike timing dependent plasticity^16^ suggests that thus inferred functional connectivity at least in part reflects synaptic connections between neurons (see Supplementary information for an analysis that supports this connection). Model studies showed that cross-correlation based analyses are far more sensitive to excitatory connections than to inhibitory ones^12,17–19^, with possible exceptions of sparse networks^20^, or networks with high background activity^21^.

Recently, an alternative way to infer functional connectivity has been proposed based on Maximum Entropy (MaxEnt) models^22^. These models provide a conceptually completely different method to infer first order interactions between pairs of neurons. Time is discretized in bins of duration $\Delta t$. In each time bin, different neurons can fire synchronously in many possible combinations, all associated with different probabilities to occur. MaxEnt models are optimized to reproduce the probability distributions of all possible combinations of synchronously active neurons. They are designed to contain a minimum number of parameters. The most often used Maximum Entropy model estimates these probability distributions using only two main parameters: a vector which encodes mean firing rates of all (N) neurons and a (NxN) matrix that describes first order interactions between all pairs of neurons^23^. The parameters describing the first order interaction between pairs of neurons could be interpreted as functional connectivity. Fitting these models has been difficult, but recent advances in machine learning allow for computationally efficient fitting of large populations of neurons^24–26^. However, it is not clear yet how this connectivity measure relates to more traditional ones that directly assess statistical correlation between pairs of neurons. The goal of the current work is to assess whether MaxEnt models can be used to estimate functional connectivity. A possible advantage of MaxEnt models is that they are in principle able to infer inhibitory as well as excitatory functional connections. Furthermore, if one uses a stimulus-dependent Maximum Entropy model^27^, it is possible to infer functional connections even during stimulation.

Here, we report on the applicability of MaxEnt models to infer functional connectivity in neural networks and possible connectivity changes induced by external stimulation. We derive a theoretical connection between functional connectivity as inferred by Conditional Firing Probabilities and MaxEnt models. We use experimental data recorded from in vitro cultures to investigate MaxEnt functional connectivity. We apply CFP and MaxEnt models to spontaneous recordings, and compare the sets of detected connections, as well as connection strengths. In addition, we stimulate cultures and compare stimulus-induced connectivity changes as detected by either method.

## Methods

### Cell culturing and transfection

Cortical cells were obtained from newborn rats. Following trypsin treatment, cells were dissociated by trituration. About 50,000 dissociated cells ( 50 $\upmu$l suspension) were plated on a multi electrode array (MEA; Multi Channel Systems, Reutlingen, Germany), precoated with poly ethylene imine (PEI). We used MEAs containing 60 titanium nitride electrodes (diameter: 30 $\upmu$m diameter; pitch: 200 $\upmu$m). Cell cultures were placed in a circular chamber (diameter: 20 mm), glued on top of the MEA. The culture chamber was filled with 1 ml of R12 medium^28^. MEAs were stored in an incubator, under standard conditions of 36 degrees C, high humidity, and $5\%$ $\mathrm {CO_2}$ in air. Culture medium was refreshed twice a week by withdrawing 500 $\upmu$l of the old medium and adding 550 $\upmu$l of fresh medium, thus compensating for evaporation. All cultures were grown for at least 3 weeks before experiments started, to allow for network maturation^15,29,30^. For recordings, we firmly sealed the culture chambers with watertight but $\mathrm {O_2}$ and $\mathrm {CO_2}$ permeable foil (MCS; ALA scientific), and placed the cultures in a measurement setup outside the incubator. In this setup, high humidity and $5\%$ $\mathrm {CO_2}$ were maintained. Recordings began after an accommodation period of at least 15 min. After the measurements, the cultures were returned to the incubator.

To enable optogenetic stimulation, cells were transfected with an adeno associated virus (AAV, serotype 2.1), obtained from Penn Vector Core, Philadelphia, Pennsylvania, USA. This virus contained the ChannelRhodopsin-2 gene, driven by the CaMKII$\alpha$ promoter, which is found exclusively in excitatory neurons. The ChannelRhodopsin-2 gene contains a mutation (H134R) which makes it sensitive for blue light 470 nm^31^. In this way the virus enables direct optogenetic stimulation of excitatory neurons, which, in turn, can activate other neurons in the network, including inhibitory ones. The initial volume of virus with a physical titre of $\approx 1.31 *10^{13}$ GC/ml was diluted 100 times in DPBS, and cultures were transduced with 25 $\upmu$l or 50 $\upmu$l the day after plating. Effective transduction was verified by the co-expression of the red fluorescent protein mCherry. All surgical and experimental procedures were approved by the Dutch committee on animal use (Centrale Commissie Dierproeven; AVD110002016802), and complied with Dutch and European laws and guidelines. Results are presented in compliance with the ARRIVE guidelines.

### Recording set-up

MEA were placed in a set-up outside the incubator to record activity. We used a MC1060BC preamplifier and FA60s filter amplifier (both MultiChannelSystems GmbH, Reutlingen, Germany). The set-up acquired signals from 59 electrodes at a sampling frequency of 16 kHz, using a custom-made Lab-View program. All analogue signals were band-pass filtered (2nd order Butterworth 0.1 to 6 kHz) before sampling. Due to their size, recording electrodes might record activity from one or more neurons. Spikes were detected whenever signals exceeded a detection threshold, set at 5.5 times the estimated root-mean-square noise level (ranging from 3 to 5 µV). for each electrode the noise estimation was continuously updated during recordings. For each threshold crossing a time stamp and electrode number were stored, as well as the waveform (6 ms). These waveforms were used for off-line artifact detection and removal using an algorithm adapted from^32^. We did not apply spike sorting. The reliability of this waveshape based method is not undisputed, as the shapes of action potentials from individual neurons can for instance substantially change during intense firing during bursts^33,34^. Thus, we used small groups of neurons as unit sources of activity, rather than individual neurons^5^.

### Stimulation of neuronal cultures

**Figure 1:**
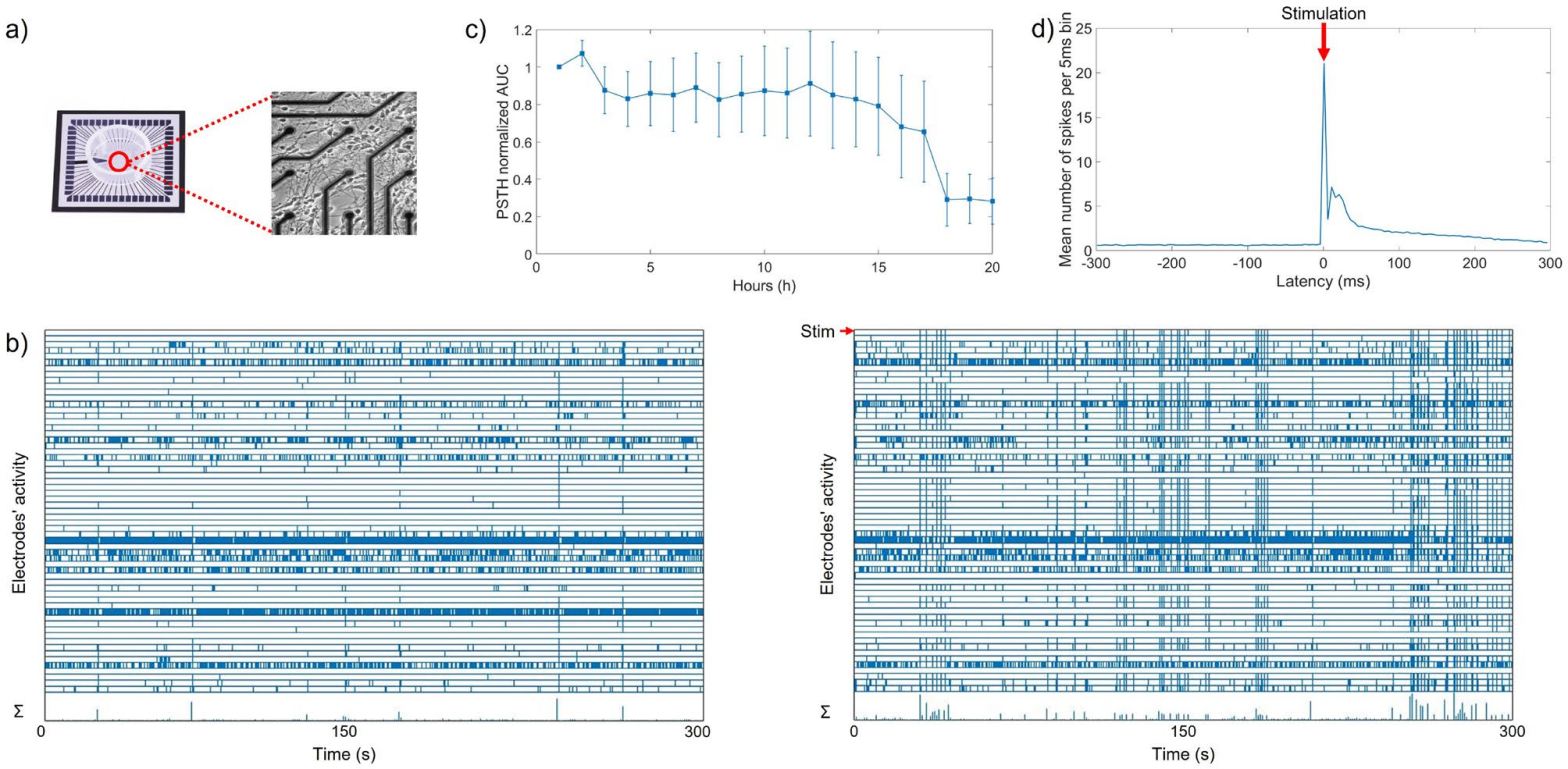
Micro electrode arrays (MEA), recorded activity and post stimulus response. Panel (**a**) shows an example of MEA with a zoom on a section of recording electrodes surrounded by several neurons. (**b**) Shows raster plots of 5 min of spontaneous activity before stimulation (left) and 5 min of activity recorded during electrical stimulation (right) Bottom pannels of both plots show summed activity in 1s bins ($\Sigma$), top panels indicate stimulation (**c**) Time course of the area under the curve (AUC) of the post stimulus histogram (PSTH) averaged across experiments. Here AUC was normalized to the first stimulation hour, given that $75\%$ of all hours were normally distributed error bars indicate SEM. (**d**) example of the averaged post stimulus response during 1 h of stimulation. Horizontal axis shows latency respective to the time of stimulation. Vertical axis shows network wide counted action potentials in 5ms bins.

Two different modes of stimulation were applied: electrical and optogenetic. Each experiment included a stimulation period of 20 h, and 1 h of spontaneous activity before and after the stimulation period. For the electrical stimulation we applied biphasic rectangular current pulses of 200 $\upmu$s per phase^5^. Current pulses were sent to one of the electrodes with interstimulus intervals taken from a fixed distribution, ranging 1–166.13 s. Possible loss of responsiveness to electrical stimulation during the 20 h experiments was evaluated by calculating the area under the curve (AUC) of the averaged post stimulus time histogram (PSTH; 15ms < latency < 300 ms), and then subtract the AUC in the interval $-300<$ latency < 0. Values were obtained for every hour (see Fig. 1c). After probing all electrodes at various amplitudes (16−24 µA), one electrode was selected for stimulation at the lowest amplitude that allowed for more than $50\%$ of the stimuli to trigger a network response. Amplitudes were low enough to avoid electrolysis.

For optogenetic stimulation, power LEDs on a SinkPAD-II 20 mm Star Base (Blue (470 nm)-74 lm@700 mA from LuxeonStarLEDs) were placed approximately 7 cm above the top of the MEA. between the LED and the MEA we placed a Faraday cage, created by a stainless steel mesh to reduce electrically induced artefacts by the LED power cables^31^. The duration and intensity of light pulses was set to induce a network response with a reliability of at least $50\%$ (typically intensity 2.5 klx, pulsewidth 100 ms). Interstimulus intervals were the same as with electrical stimulation.

### Experimental design

In total, 34 cultures were used in this study. All cultures were tested for activity and stimulus responses before experiments started, and cultures with less than ten active electrodes or without a clear responses to stimulation (example in Fig. 1) were not used for experiments.

#### Influence of inactive electrodes on MaxEnt

In MEA recordings, some of the electrodes may record only few ($<250$ per hour), or no action potentials (e.g. if there are no cells on the electrode), these are referred to as inactive electrodes. Cross-correlation based methods to infer functional connectivity typically do not take these electrodes into account. Maximum Entropy (MaxEnt) models, however, do not discriminate between active and inactive electrodes, which might affect inferred connectivity. To determine whether and how the inclusion of inactive electrodes affected MaxEnt connectivity estimates, we used 20 recordings of 1 h of spontaneous activity. We first inferred functional connectivity using MaxEnt on all data, including the inactive electrodes, and then repeated the procedure on the subset of active electrodes. We determined the correlation between the J matrices as obtained in both approaches, considering only the connections between active electrodes (see Supplementary information for results).

#### Relating CFP and MaxEnt

We first determined a mathematical relationship between functional connectivity inferred by CFP (*M*) and MaxEnt (*J*). As *J* is symmetric by definition and *M* is not, we related $M_{i,j} + M_{j,i}$ to $J_{i,j} + J_{j,i}$. We used 20 1 h recordings of spontaneous activity to verify this relationship. We calculated the *M* and *J*, and used the derived relationship (Eq. 6) to calculate ${\hat{J}}$. We determined correlation coefficients between ${\hat{J}}$ and the actual J as obtained by MaxEnt, taking into account only the non-zero positions of the *M* matrix.

#### Quantification of spontaneous functional connectivity changes on long recordings

We used 4 long ($\approx$ 20 h) spontaneous activity recordings to quantify spontaneously occurring connectivity changes, as inferred by MaxEnt and CFP,Both models were applied to 1 h chunks of data (hours 1–5, 10, 15, and 19), taking into account only electrodes that were active ($>250$ action potentials) in all chunks. We calculated the fraction of excitatory and inhibitory connections during the first 5 h, to enable comparison to work by others^21^ We determined Euclidean distances (Eq. 4) between these connectivity matrices ($J_n$, $n=2,3,4,5,10,15,19$) and that of the first hour, $J_1$. We also calculated similarity indices (Eq. 5) between sets of excitatory and inhibitory connections during these hours.

#### Connectivity changes

Cultures were stimulated (electrically ($n=10$) or optogenetically ($n=10$)) to investigate the effect on connectivity as estimated by CFP and MaxEnt. CFP and MaxEnt were fitted to 1 h chunks of spontaneous activity collected before (Baseline) and after the 20 h stimulation period (AftStim). Both Baseline and AftStim recordings were divided into two blocks of 30 min. First, the Euclidean distance was calculated between both connectivity matrices within Baseline. Then, Euclidean distances were calculated between both AftStim connectivity matrices and both Baseline matrices, and averaged. Only electrodes that were active during Baseline and AftStim were taken into account. We checked for possible differences between Euclidean distances within Baseline with the ones induced by the stimulation.

### Assessing functional connectivity

#### Conditional firing probability

Conditional Firing Probability (CFP) models estimate the probability that neuron *j* fires at t=$\tau$ ($0 \le \tau < 500$ ms), given that neuron *i* fired at t=0. Only active electrodes were used in this analysis. All electrodes that recorded $>250$ spikes in a period of 1 hour were considered to be active. For each pair of active electrodes (*i*, *j*) the obtained histograms (0.5 ms bin size) are fit by the equation^15^

1 $$\begin{aligned} CFP_{ij}(\tau ) \approx o_{i,j} + \frac{M_{i,j}}{1+\left( \frac{\tau -T_{i,j}}{w_{i,j}}\right) ^2}. \end{aligned}$$

In this equation $M_{i,j}$ is interpreted as the strength of the connection, $T_{i,j}$ as the latency. $o_{i,j}$ represents uncorrelated background activity and $w_{i,j}$ accounts for the width of the peak. Values were estimated by minimizing the summed squared error using a Nelder-Mead simplex algorithm. The bin size influences the values of *M* and *o*, as these are related to the probability to record a spike during a time window of that size. *T* and *w* are timing dependent parameters. If this standard function could not be fitted properly, resulting in $w_{i,j} >250ms$, $T_{i,j} >250ms$, or $M_{i,j} \le o_{i,j}$, the strength of the connection was set to $M_{i,j}=0$.

#### Maximum entropy models

In the most popular Maximum Entropy model, neuronal activity between time *t* and $t+\Delta t$ are assessed and recorded in a binary vector $\vec{\sigma }$. We set $\Delta t$ to 100 ms. If neuron *i* fires in that time frame, $\sigma _i$ is set to 1, otherwise, $\sigma _i=0$. After construction of the binary vectors their probability distributions are modeled as

2 $$\begin{aligned} p(\vec{\sigma }) = \frac{1}{Z} e^{\left( -\theta ^{\top }\vec{\sigma } + \vec{\sigma }^{\top } J \vec{\sigma }\right) } \end{aligned}$$

where *Z*, the partition function, is a normalization factor:

3 $$\begin{aligned} Z = \sum _{\vec{\sigma }}e^{\left( -\theta ^{\top }\vec{\sigma } + \vec{\sigma }^{\top } J \vec{\sigma }\right) }. \end{aligned}$$

**Figure 2:**
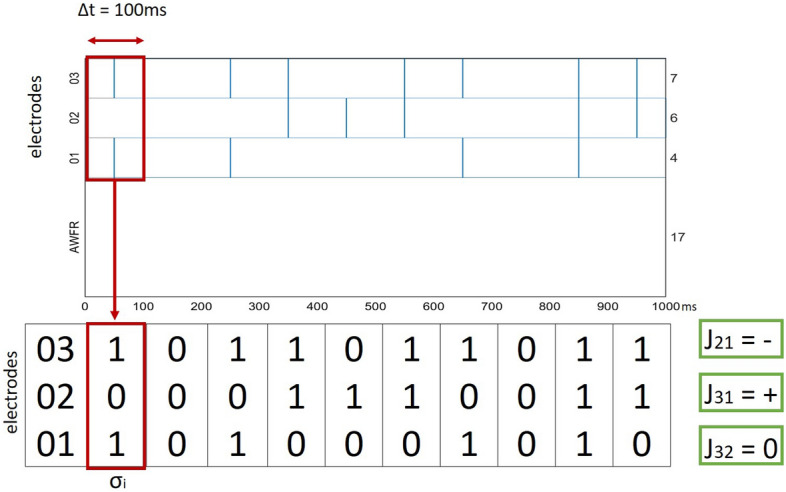
Visualization of the MaxEnt $J_{ij}$. Top graph shows simulated activity recorded from 3 electrodes during a period of 1 *s*. Left vertical axis shows electrodes numbers and stimulation (empty; spontaneous activity). Right vertical axis shows total number of recorded spikes per electrode. Horizontal axis: time in *ms*. Bottom panel shows the binary vectors $\vec{\sigma _{i}}$ calculated using a $\Delta t$ of 100 *ms*. From the binary vectors it is then possible to estimate the nature of the different $J_{ij}$. $J_{ij}$ is negative if the two electrodes have an inhibitory connection, positive if the connection is excitatory and 0 if there is no connection.

In Eq. (3) ${\theta }$ is a vector that represents the propensity of neurons to fire, and *J* represents first order interactions between neurons. *J* is symmetric by definition, and is considered to describe functional connectivity between neuron *i* and *j*. The values of *J* can be positive, indicating an excitatory connection, negative, indicating an inhibitory connection, or 0, indicating no connection, see Fig. 2. The most difficult aspect of using the Maximum Entropy model is that it was hard, until recently, to infer $\theta$ and *J*. We use a somewhat recently developed technique called Minimum Probability Flow (MPF)^24^ to infer these parameters. An analysis of the goodness of fit of MPF is given in the Supplementary Information. Although there is good reason to suspect that the parameters inferred by MPF are not the parameters that cause the best match between model and data, the parameters inferred by MPF seem to still yield accurate assessments of functional connectivity, and detect whether or not connectivity has changed over the course of the experiment (see Supplementary information).

### Readouts for connectivity changes

#### Euclidean distances

Spontaneously occurring or stimulus-induced changes in network connectivity were quantified by the Euclidean distance (ED) between two connectivity matrices *S*. Here *S* represents a general matrix, referring to the connectivity matrix *M* when CFP is applied, and to the matrix *J* when MaxEnt is used instead.

4 $$\begin{aligned} ED(t) = \sqrt{\sum \limits _{i=1}^n \sum \limits _{j=1}^n (S_{ij}(t) - S_{ij}(t_{0}))^2}, \end{aligned}$$

where $t > t_{0}$. Only nonzero values present in both connectivity matrices were considered.

#### Similarity index

Similarity between the sets of excitatory (*E*) and inhibitory (*I*) connections were assessed calculating a similarity index (*SI*) expressed as following^15^:

5 $$\begin{aligned} SI_{E/I} = \sqrt{\frac{|(Ja=E,I)\cap (Jb=E,I)|^2}{|(Ja=E,I)| \cdot |(Jb=E,I)|}}. \end{aligned}$$

Here *Ja* indicates the set of either excitatory or inhibitory connections in matrix *Ja*, while *Jb* indicates that set for matrix *Jb*. ${\Vert }$ are used to obtain set sizes; $|(Ja=E,I)\cap (Jb=E,I)|$ indicates the number of connections that are excitatory in *Ja* and *Jb*. SI was also calculated between M matrices, for excitatory connections only.

### Statistical analysis

Normality of distributions was assessed by a Shapiro-Wilk test. In case of normality, group means +/- standard error of the mean (SEM) are presented and group means were compared by a Student t-Test. Otherwise, median values and $32\%$-$68\%$ percentiles are shown, and the Mann-Whitney test was used. P-values $<0.05$ were considered to indicate significant differences. All statistical analysis were performed using SPSS statistics for Windows (IBM, Inc., Chicago, IL) or Matlab (The Mathworks, Inc., Natick, MA, USA).

## Results

Cultures had on average, $32.4 \pm 2.1$ (out of 59) active electrodes, with a mean firing rate of $1.5 \pm 1.2$ spikes/s/electrode (see Table 1 ). MaxEnt connectivity based on all electrodes was very similar to that based on active electrodes only, with a mean correlation coefficient of $0.87 \pm 0.02$ (see Supplementary information). To have a fair comparison with CFP connectivity, all further analyses were performed on active electrodes only. We first reveal the theoretical relationship between CFP and MaxEnt and verify this using spontaneous recordings. Then, we analyze spontaneous connectivity fluctuations in long recordings as quantified by MaxEnt and CFP models.

Finally, we show how both methods detect large connectivity changes induced by electrical stimulation, and much smaller changes by optogenetic stimulation.

**Table 1:** Characteristics of networks used in experiments.

Type of experiment	Number of experiments	Mean number of active electrodes (baseline)	Mean number of total spikes (baseline)	Mean number of total spikes (first hour of stimulation)	Mean number of total spikes (after stimulation)
Influence of inactive electrodes and correlation CFP and MaxEnt	20	31.5 ± 12.9	133046 ± 113969.4	Does not apply	Does not apply
Stability of CFP and MaxEnt	4	17.5 ± 5.7	73190.8 ± 24371.3	Does not apply	Does not apply
Electrical stimulation	10	37.3 ± 8.2	183203.3 ± 97969.3	258234.2 ± 141886.2	90041 ± 58038.5
Optogenetic stimulation	10	35.2 ± 10.7	298843.6 ± 177255.2	341378.5 ± 156726.1	178368.3 ± 153222.6

Second column shows the number of cultures used per type of experiment, third column shows the number of active electrodes in the first hour of spontaneous activity (mean ± SD). The three columns on the right show the number of action potentials recorded during baseline (1 h), the first hour of stimulation, and 1 h of spontaneous activity after stimulation (mean ± SD).

### Relationship between CFP and MaxEnt functional connectivity

**Figure 3:**
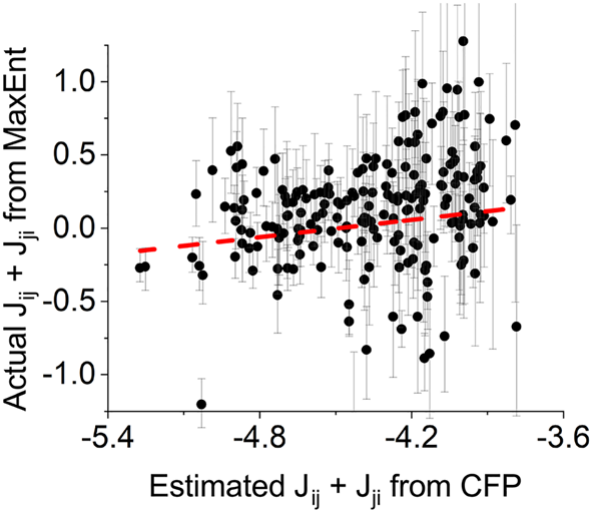
Relationship between directly inferred MaxEnt connectivity *J* and MaxEnt connectivity estimated from CFP parameters ${\hat{J}}$. Both models were applied to spontaneous activity of n=20 cultures. *x*-axis shows the predicted ${\hat{J}}_{ij}+{\hat{J}}_{ji}$ from Eq. (6), while the *y*-axis shows the directly inferred estimates $J_{ij}+J_{ji}$ of those connections that were found by CFP analysis (R= 0.44). Red dashed line shows fitted linear trend. Error bars indicate $32$–$68\%$ percentiles.

We derived a theoretical relationship between CFP and MaxEnt. The key is to compute $P(\sigma _j(t+\tau )=1|\sigma _i(t)=1)$, or $CFP_{ij}(\tau )$, assuming that the MaxEnt model is correct, and thereby relate the CFP connectivity matrix *M* to the MaxEnt connectivity matrix *J*. The obtained relation solves as (see Supplementary information for detailed mathematical derivation)

6 $$\begin{aligned} {\hat{J}}_{ij}+{\hat{J}}_{ji}&= \frac{1}{2}\log \left( \frac{1}{2} \frac{1}{\lambda _i\lambda _j} \left( \frac{1}{2} \lambda _i \left( o_{ij} + M_{ij} \frac{w_{ij}^2}{w_{ij}^2+T_{ij}^2}\right) +\frac{1}{2} \lambda _j \left( o_{ji} + M_{ji} \frac{w_{ji}^2}{w_{ji}^2+T_{ji}^2}\right) \right) \right) , \end{aligned}$$

plus corrections of $O(\Delta t)$. Here, ${\hat{J}}$ represents the predicted MaxEnt connectivity, calculated from the CFP connectivity, and $\lambda$ represents the mean firing rate. We verified this relation on experimental data, inferring functional connectivity with both CFP and MaxEnt. Figure 3 shows an example of inferred MaxEnt connectivity (*J*) versus MaxEnt connectivity estimated from CFP (${\hat{J}}$) using Eq. (6). In 20 recordings the correlation coefficient between $J_{ij}+J_{ji}$ and ${\hat{J}}_{ij}+{\hat{J}}_{ji}$ averaged $0.32 \pm 0.03$.

Despite the value shift between directly inferred $J_{ij}+J_{ji}$ and that derived from CFP-estimated functional connectivity, there was a moderate correlation between connection strengths as obtained by both methods. The time needed to compute CFP and MaxEnt connectivity matrices increased with increasing number of active electrodes and with the increasing number of recorded spikes. When analyzing>40 active electrodes or >150000 spikes per hour, MaxEnt was computationally more efficient than CFP (see Supplementary Information, Fig. S2).

### Quantification of spontaneous functional connectivity changes on long recordings

**Figure 4:**
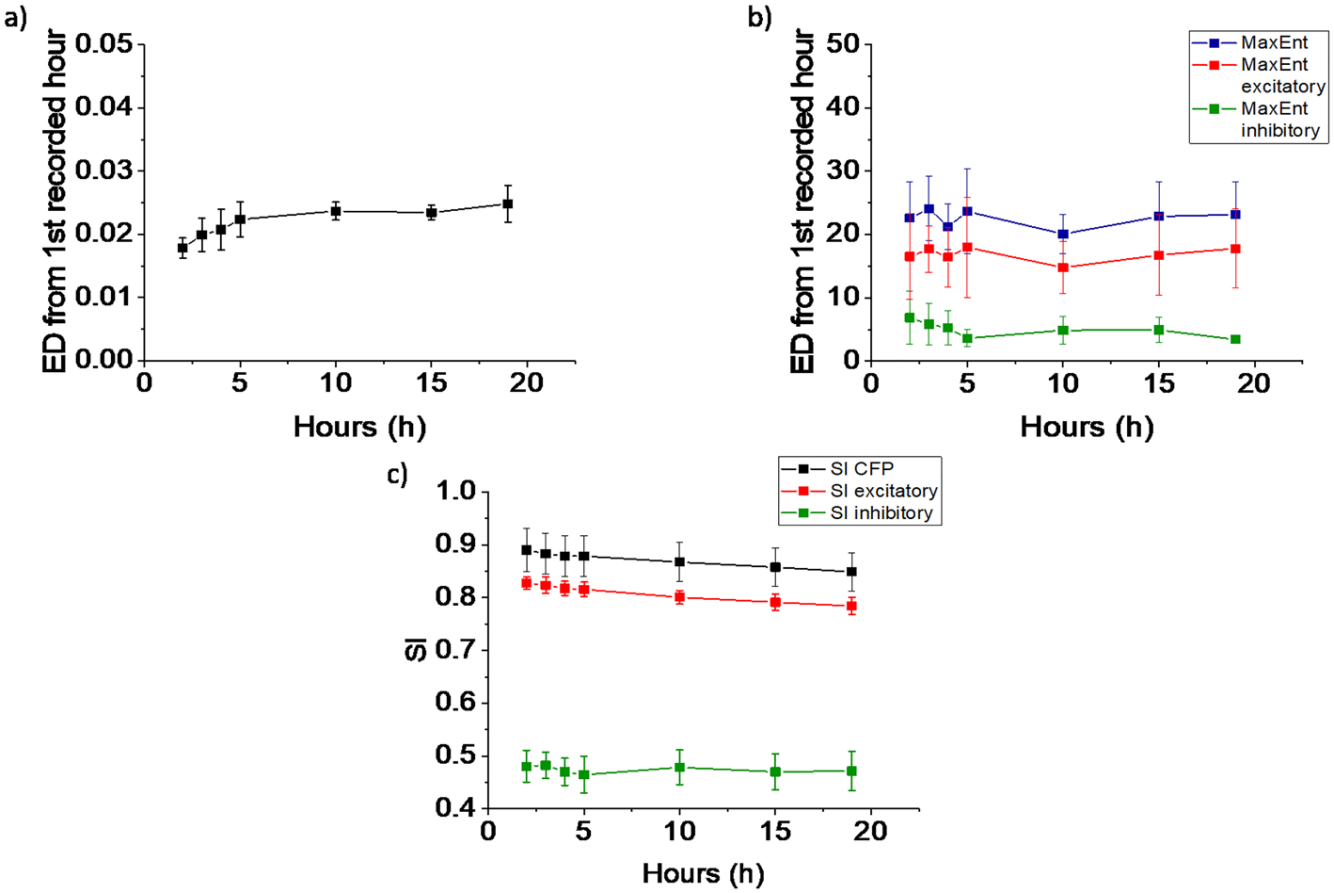
Inferred connectivity from spontaneous activity recordings using CFP or MaxEnt models during time. (**a**) CFP connectivity matrices were calculated for every hour of recording. Shown are mean Euclidean distances to the connectivity matrix of the first hour (n = 4 networks). CFP connections slowly drift away from the starting connectivity. (**b**) Same analysis as in (**a**) but with MaxEnt connectivity matrices, all connections (blue), excitatory connections only (red) and inhibitory connections only (green). Results show a similar trend as for CFP connectivity (**c**) Similarity indices (SI) of excitatory (red) and inhibitory (green) connections of MaxEnt connectivity matrices, and similarity indices of CFP connectivity matrices (black). around $85\%$ of CFP connections (mainly excitatory) and $80\%$ of MaxEnt excitatory connections remained unchanged. On the other hand around $50\%$ of MaxEnt inhibitory connections seemed to not change during long recordings. Error bars indicate SEM, and refer to differences between networks.

Figure 4a shows that CFP connectivity (connectivity matrix *M* values range up to 0.02) in 19 h recordings exhibits slow drift away from the initial connectivity. The set of connections was very stable, with $>85\%$ of connections unchanged, in agreement with earlier work^15^. MaxEnt connectivity contained excitatory as well as inhibitory connections (connectivity matrix *J* absolute values range up to 21). During the first 5 h of these recordings, on average $74\% \pm 4\%$ of all connections were excitatory, and $26\% \pm 4\%$ were inhibitory. Both sets of connections showed a similar drift as seen with CFP connectivity, see Fig. 4b. The set of excitatory connections in MaxEnt connectivity was very stable, with more than $80\%$ of connections unchanged, while $\approx$ $50\%$ of all inhibitory connections persisted.

### Connectivity changes induced by stimulation

We investigated changes in functional connectivity induced by electrical or optogenetic stimulation on, as inferred by MaxEnt and CFP. Connectivity changes were quantified by the Euclidean distances between connectivity matrices before and after stimulation. Connectivity fluctuations within baseline did not significantly differ between cultures that received electrical stimulation or optogenetic stimulation (CFP $p = 0.17$, MaxEnt $p = 0.77$).

**Figure 5:**
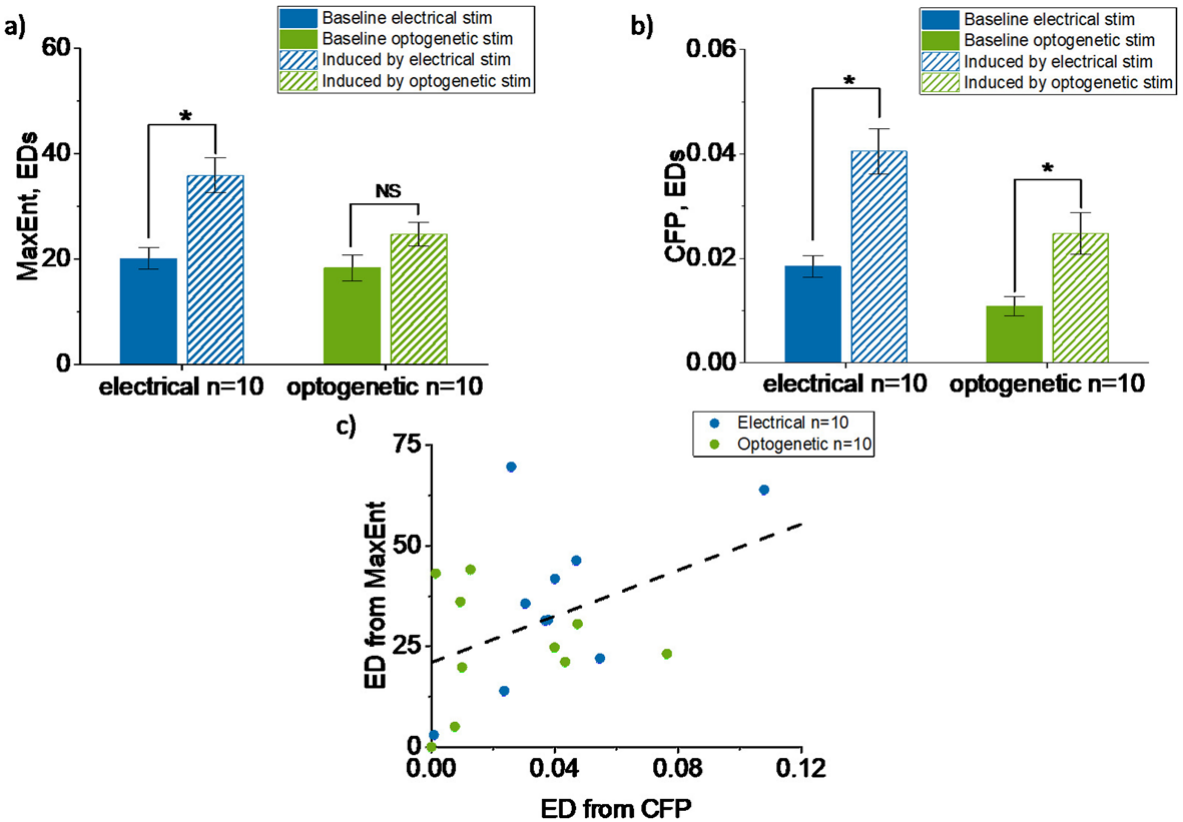
Connectivity changes induced by electrical (blue) and optogenetic stimulation (green). Euclidean distances were calculated first within baseline recordings, and then between the connectivity matrices before and after a 20 h stimulation period. (**a**) Comparison between connectivity changes within baseline with the ones induced by the different stimulation modalities, obtained from MaxEnt (electrical $p = 0.004$, optogenetic $p = 0.16$). (**b**) Same as panel A but results obtained using CFP (electrical $p = 0.02$, optogenetic $p = 0.01$). Error bars indicate SEM. (**c**) ED calculated based on all connections in CFP connectivity matrices ($ED_{CFP}$) and MaxEnt matrices ($ED_{MaxEnt}$). Correlation coefficient: $R = 0.42$. Black dashed line shows fitted linear trend.

CFP detected signifcant stimulation-induced connectivity changes (electrical stimulation: $p = 0.02$; optogenetic stimulation: $p = 0.01$). MaxEnt also detected significant connectivity changes in response to electrical stimulation ($p = 0.004$), but not to optogenetic stimulation ($p = 0.16$). Both methods yielded larger connectivity changes after electrical stimulation than after optogenetic stimulation (Fig. 5).

All MaxEnt and CFP Euclidean distances induced by the stimulation were normally distributed (CFP electrical $p = 0.07$, CFP optogenetic $p = 0.07$, MaxEnt electrical $p = 0.92$, MaxEnt optogenetic $p = 0.61$). Euclidean distances within baseline were normally distributed for MaxEnt (both electrical and optogenetic stimulation recordings) and CFP electrical stimulation data, but not for CFP optogenetic stimulation data (CFP electrical $p = 0.61$, CFP optogenetic $p = 0.02$, MaxEnt electrical $p = 0.19$, MaxEnt optogenetic $p = 0.38$).

We then grouped all stimulation experiments. Connectivity changes as detected by MaxEnt, including both excitatory and inhibitory connections, and CFP showed a correlation coefficient of $R = 0.42$. (See Fig. 5c). A comparable correlation coefficient with CFP, was obtained when considering only excitatory connections in MaxEnt ($R = 0.47$).

## Discussion

Cross-correlation based methods have been commonly used to infer functional connectivity in neuronal networks, despite drawbacks like their relative insensitivity to inhibitory connections and difficulties to infer connectivity during stimulation. Recently, coupling constants of Maximum Entropy (MaxEnt) models have been proposed to describe functional network connectivity^23^. This is a conceptually completely different approach, that optimizes two variables—a vector that describes the probability to fire for all neurons, and a matrix of coupling constants that account for pair-wise first order interactions between neurons—to approximate the probability distributions of all possible combinations of neurons to fire in synchrony^23^. We used data from in vitro neuronal networks to show that MaxEnt models provide connectivity estimates that correlate well with those obtained by conditional firing probabilities (CFP), an established cross-correlation based method^15^. In addition, MaxEnt models were able to detect stimulus-induced connectivity changes, which were of the same magnitude as those detected by CFP.

Earlier work showed that CFP estimates of connectivity in mature, unstimulated network present mainly minor fluctuations during periods of 1 day^15^,and that stimulation at one electrode induced significantly larger connectivity changes^15^. Comparable results were obtained in the current study, where spontaneously occurring connectivity changes detected by CFP during 19 h seem to be smaller than those induced by electrical stimulation, and showed no clear trend away from baseline connectivity. MaxEnt models yielded very similar spontaneous connectivity fluctuations, which appeared to be smaller than stimulus-induced connectivity changes.The vast majority of all excitatory connections persisted during 19 h without stimulation. These results indicate that MaxEnt models thus provide stable estimates of excitatory connectivity during at least 19 h. MaxEnt Euclidean distances were several orders of magnitude larger than CFP distances. This probably reflects the different ranges of connectivity strengths in both methods, rather than different sensitivity to detect stimulus induced connectivity changes. Inhibitory connectivity was revealed only by MaxEnt models, and appeared less stable (Fig. 4). This may reflect methodological difficulties to detect inhibitory connections, or may reveal inherent different stability between inhibitory and excitatory connections. Recently, two methods were developed that are, in principle, able to detect inhibitory connections. Both methods, however, impose additional requirements on networks (sparseness)^20^ or recorded activity ($\ge 6$ spikes/s/electrode)^21^. The first method was validated only on computational models, and it is not fully clear how observed limitations (low density random networks) translate to our biological networks. Still with this method inhibition was more difficult to identify than excitation^20^. Our data did not show the relatively high level of ongoing activity required for the second method. Still, the current finding that $26\% \pm 4\%$ of all connections were inhibitory is in good agreement with their observation that $\approx 25\%$ of all connections were inhibitory^21^.

In MaxEnt models the sign of a connection reflects whether the connection is excitatory or inhibitory, but the absolute value cannot be interpreted as straightforwardly as in CFP models, which obscures direct comparison of both methods. To that end, we derived and verified a theoretical connection between MaxEnt and CFP-based functional connectivity. Even though the computationally-efficient Minimum Probability Flow does not appear to fit the Maximum Entropy model well (see Supplementary information), the two methods showed a moderate correlation. This is remarkable because the relationship between the differently inferred connectivities is highly non-linear, and contains factors that depend on specific properties of individual cultures. The moderate correlation coefficients emphasize that both conceptually different methods to infer connectivity tended to yield the same set of connections, with similarly distributed strengths across this set.

Mismatches between estimated and true values of $J_{ij}+J_{ji}$ may come from errors in the approximation (e.g. due to a large value of $\frac{J_{ij}+J_{ji}}{\min (|\theta _i|,|\theta _j|)}$), and errors in parameter inference. This might happen either due to an inability of Minimum Probability Flow to infer correct parameters when the model is out-of-class^24^, meaning that the data are derived from a distribution that does not match the model to which the data is fit, or due to the fact that these models tend to be “sloppy”^35^. Sloppy models have hard-to-infer parameters, such that a wide set of parameters yield similar model predictions (see Supplementary information). Improvements to parameter inference via changes to hyperparameters in Minimum Probability Flow will likely lead to increases in correlation between the two functional connectivity methods, as the parameters inferred for the Maximum Entropy models will be closer to the true parameters that would best match the fit between data and model.

Finally, we investigated whether MaxEnt models provide good estimates of the efficacy of electrical versus optogenetic stimulation in changing functional connectivity. Earlier work, using CFP analysis, showed that repeated electrical stimulation through one electrode induces connectivity changes in cultured neuronal networks, but stimulation through randomly changing electrodes had no effect on connectivity^7^. Activity and connectivity mutually affect each other, and it has been hypothesized that networks develop an equilibrium between activity and connectivity. It requires repeated activation of patterns not included in the spontaneous activity repertoire to drive networks out of this equilibrium^4^, and the driving force applied by random stimulation is apparently insufficient to achieve this. In principle optogenetic stimulation does not directly activate inhibitory neurons, which have been described to have an important hub function in developing networks^36^. Activation of hub neurons might be crucial to induce connectivity changes. However, inhibitory neurons did become activated indirectly in this stimulation modality. It thus seems unlikely that activation of inhibitory hub neurons was the critical difference between electrical and optogenetic stimulation. As an alternative, we hypothesize that optogenetic stimulation probably lacks specific new patterns, and therefore does not exert a driving force towards a new equilibrium. Accordingly, electrical stimulation induced significant changes in both MaxEnt and CFP connectivity. Optogenetic stimulation induced changes were significant only in CFP connectivity. Still, the magnitudes of induced MaxEnt and CFP connectivity changes were well-correlated, and both methods showed the same trend of smaller connectivity changes upon optogenetic stimulation. The power of our analysis would increase if future studies would use the same cultures for electrical and optogenetic stimulation. Even though there were no significant differences in spontaneous connectivity fluctuations between the cultures used for the two stimulation modalities, this protocol modification would exclude possible culture-specific differences.

In conclusion, MaxEnt models can be applied to infer functional connectivity based on activity recorded from in vitro neuronal networks. They provide a stable measure of connectivity and detect stimulus induced connectivity changes. Inferred connectivity and the magnitude of stimulus-induced changes correlated well to those inferred by CFP, a cross-correlation based method. Although strengths of MaxEnt connectivity are less straight-forward to interpret than those inferred by CFP, they discriminate between excitatory and inhibitory connections. Inhibitory connections appeared less stable than excitatory ones, but we cannot exclude that this reflects methodological difficulties to infer inhibitory connectivity. Thus, MaxEnt models provide a suitable alternative to cross-correlation based methods to infer excitatory functional connectivity. This, together with the potential benefit of being able to estimate functional connectivity in the presence of a stimulus^27^, make a potentially powerful new tool to study living neuronal network connectivity.

## Supplementary Information

Supplementary Information.

## Data Availability

Data files are available from Dryad (https://doi.org/10.5061/dryad.p5hqbzkqj). Additional information related to experimental conditions, data formats, etc. is available on request. Please contact the corresponding author.
