# Author Correction: Maximum entropy models provide functional connectivity estimates in neural networks

**DOI:** 10.1038/s41598-022-17289-7

**Published:** 2022-07-26

**Authors:** Martina Lamberti, Michael Hess, Inês Dias, Michel van Putten, Joost le Feber, Sarah Marzen

**Affiliations:** 1 Department of Clinical Neurophysiology, University of Twente, P.O. Box 217, 7500 AE Enschede, The Netherlands; 2 Laney Graduate School, Emory University, Atlanta, GA 30307 USA; 3 W. M. Keck Science Department, Pitzer, Scripps and Claremont McKenna College, Claremont, 91711 USA; 4 Clinical Neurophysiology TechMed Building, TL 3382, University of Twente, P.O. Box 217, 7500 AE Enschede, The Netherlands

Correction to: *Scientific Reports* 10.1038/s41598-022-13674-4, published online 10 June 2022

The Acknowledgments section in the original version of this Article was incomplete.

“The authors thank dr Gerco Hassink and Marloes Levers for the technical assistance in cell culture preparation. This study was supported by the US Air Force Office for Scientific Research, Grant Number FA9550-19-1-0411.”

now reads:

“The authors thank Dr. Gerco Hassink and Marloes Levers for the technical assistance in cell culture preparation. We also thank Christopher Hillar for personal communications and the use of his open source software package https://github.com/team-hdnet/hdnet. This study was supported by the US Air Force Office for Scientific Research, Grant Number FA9550-19-1-0411.”

As a result of this error, reference 26 was omitted from the text and the reference list.

“The parameters describing the first order interaction between pairs of neurons could be interpreted as functional connectivity. Fitting these models has been difficult, but recent advances in machine learning allow for computationally efficient fitting of large populations of neurons^24,25^.”

now reads:

“The parameters describing the first order interaction between pairs of neurons could be interpreted as functional connectivity. Fitting these models has been difficult, but recent advances in machine learning allow for computationally efficient fitting of large populations of neurons^24,25,26^.”

All the subsequent references have been renumbered.

The original Article has been corrected.

