# Supplementary material for: Maximum entropy models provide functional connectivity estimates in neural networks
Source: Sci Rep. 2022 Jun 10;12:9656. doi: 10.1038/s41598-022-13674-4 (PMC9187636; doi:10.1038/s41598-022-13674-4)
Supplement: Supplementary file 1 — Supplementary Information. [file 41598_2022_13674_MOESM1_ESM.pdf]

Maximum Entropy models provide functional connectivity estimates in neural networks

Martina Lamberti[1], Michael Hess[2, 3], Inês Dias[1], Michel van Putten[1], Joost le Feber[1,+,*], and Sarah Marzen[3,+]

[1]Department of Clinical Neurophysiology, University of Twente, Enschede, PO Box 217 7500AE, The Netherlands
[2]Laney Graduate School, Emory University, Atlanta, GA 30307, USA
[3]W. M. Keck Science Department, Pitzer, Scripps and Claremont McKenna College, Claremont, 91711, USA
[+]these authors contributed equally to this work
[*]correspondence to Joost le Feber Clinical Neurophysiology University of Twente PO Box 217 7500AE Enschede Netherlands j.lefeber@utwente.nl

May 19, 2022

Supplementary information

Relating functional and statistical connectivity

Functional connectivity is not exactly the same as statistical connectivity, especially if not all neurons are observed. Then, common input from a third neuron can create or alter an apparent connection between two other neurons. But the two are certainly related. Here, we show that in a simple model of neural firing, when all neurons are observed, statistical and functional connectivity are directly related.

Our dynamical model will be that of coupled linear leaky integrate-and-fire neurons with a soft threshold for spiking.

Neuron i's membrane potential is given by V_i, and evolves according to the equation

$$\frac{dV_i}{dt} = -\frac{1}{\tau}V_i + \Theta_i + I_i(t) + \sum_j J_{ij} \sum_a g(t - t_j^{(a)}), \tag{S1}$$

which is essentially a simplified electrical circuit model of the neural activity. There are three main terms. The term $-\frac{1}{\tau}V_i + \Theta_i$ implicitly assumes that ion channel conductances are constants; the time constant τ relates to the membrane resistance and capacitance, while Θ_i depends on the equilibrium potentials and conductances associated with the various ion channels. The term $I_i(t)$ allows for current injection to affect the membrane potential; we ignore this term and set it to 0. Finally, the term $\sum_j J_{ij} \sum_a g(t - t_j^{(a)})$ allows the firing of neuron j at a time of $t_j^{(a)}$ to increase or decrease the rate of change of the membrane potential of neuron i by $J_{ij}g(t - t_j^{(a)})$, where J_{ij} is a "synaptic connectivity" and g is any function, usually chosen to be exponential. More generally, the hard threshold is replaced by a "soft threshold" in which the probability of a neuron firing in a time bin of size Δt is some function of the current membrane potential. That is, neuron i fires with rate $f(V_i - V_{th})$ for some (increasing) function f. This formulation allows for a neuron to fire even when it is below threshold. This is not necessarily biophysically reasonable, but it provides a better statistical match to Maximum Entropy models. The soft threshold leads to a hard threshold when $f(x) = \begin{cases} \infty, & \text{if } x \geq 0 \\ 0, & \text{if } x < 0 \end{cases}$.

Our next goal is to get statistics of $P(\sigma_i = 1)$ and $P(\sigma_j = 1, \sigma_i = 1)$ for the dynamical model, from which we can infer a relationship between dynamical parameters and MaxEnt parameters. Assuming stationarity, we have

$$P(\sigma_i = 1) \approx \langle f(V_i - V_{th}) \rangle \Delta t. \tag{S2}$$

We can think of this in the following way: there is some mean-field activity; neurons are firing, producing spikes of neuron i with some frequency. In general, it is not permissible to set $\langle f(V_i - V_{th}) \rangle$ to $f(\langle V_i \rangle - V_{th})$; higher-order terms might be non-negligible.

Next, we have to calculate $P(\sigma_j = 1, \sigma_i = 1)$. We can case this out into two cases, one in which neuron i fires first and one in which neuron j fires first. Roughly speaking, the weights of this happening correspond to the mean-field probabilities of them firing independently. The weighting factor is $\langle f(V_{i/j} - V_{th} + J_{ij/ji}) \rangle \Delta t$, since $e^{-\Delta t/\tau_{i/j}}$ is roughly 1 when Δt is quite small. Taylor expanding gives $\langle f(V_{i/j} - V_{th}) \rangle + J_{ij/ji} \langle \frac{d}{dV} f(V - V_{th}) \rangle + O(J^2)$. After some algebra, we find that

$$\hat{J}_{ij} + \hat{J}_{ji} \approx \log \left(1 + \frac{1}{2} \left(\frac{\langle f' \rangle_i}{\lambda_i} J_{ji} + \frac{\langle f' \rangle_j}{\lambda_j} J_{ij} \right) \right). \tag{S3}$$

When we have a hard threshold, then $\langle f' \rangle$ is simply the value of the probability density function at the threshold voltage, or the firing rate. Hence, in that case,

$$\hat{J}_{ij} + \hat{J}_{ji} \approx \log \left(1 + \frac{J_{ji} + J_{ij}}{2} \right). \tag{S4}$$

This development mirrors that of Ref.[1], but differs in that we are assuming that the time bin size Δt for the MaxEnt method is quite small. As such, we end up finding a direct relationship between the two connectivities.

Our Eq. S4 explain the previously unexplained correspondence between $\hat{J}_{ij}$ and $J_{ij} + J_{ji}$ shown in the Appendix of Ref.[1]. In practice, we have found it difficult to find a regime such that the conditions of Eq. S4 hold.

Influence of inactive electrodes on MaxEnt connectivity estimation

We calculate MaxEnt connectivity based on all electrodes (J_{all}), and based on active electrodes only (J_{active}), and compare results for connections between active electrodes. Figure S1 is a typical example, showing a relatively high correlation coefficient between J_{all} and J_{active}. All 20 analysed samples yielded similar results, with an average correlation coefficient of 0.87 ± 0.02. A closer analysis reveals that there is a strong correlation between the excitatory connections in both approaches, shown in blue in Figure S1 ($R = 0.86 \pm 0.02$), and also a strong correlation between the inhibitory connections, shown in green ($R = 0.87 \pm 0.02$). This resulted in a Euclidean distance between J_{all} and J_{active} of $\approx 8.78 \pm 1.24$, which is in line with distances between subsequent connectivity matrices obtained from continuous spontaneous recordings which were $\approx 22.52 \pm 1.95$ (see figure on spontaneous connectivity changes in main text).

Theoretical relationship between CFP and MaxEnt functional connectivity

We can relate the two statistical models. The key is to compute $P(\sigma_j(t+\tau) = 1 | \sigma_i(t) = 1)$, or $CFP_{ij}(\tau)$, assuming that the MaxEnt model is correct, and thereby relate the CFP connectivity matrix M to the MaxEnt connectivity matrix J. Our assumptions are that we are in a small-coupling limit, i.e. that the connections are weak relative to the innate propensity of each neuron to fire.

Under the CFP model, the probability that two neurons i and j end up spiking in the same time bin comes from two separate integrals that ignore the contributions of other neurons (or simply assumes that those contributions wash out).

We start by relating the probability of two neurons firing in the same time bin to the probability density of those two neurons firing:

$$P(\sigma_i = 1, \sigma_j = 1) \quad = \quad \int_0^{\Delta t} \int_0^{\Delta t} P(\sigma_i(t), \sigma_j(t')) dt dt'. \tag{S5}$$

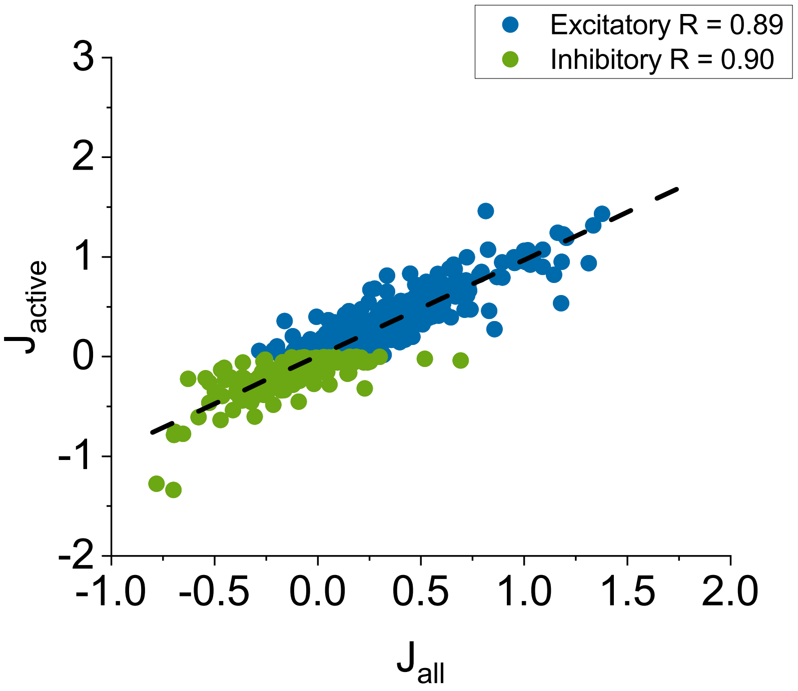

Figure S1: Example of correlation between the connectivity matrix J_{all}, obtained by including all electrodes, and J_{active} based on active electrodes only. There is a strong correlation between the strengths of excitatory connections (blue; $R = 0.89$), and between the strengths of inhibitory connections (green; $R = 0.90$). Black line shows fitted linear trend.

Either neuron i fires first, or neuron j fires first:

$$P(\sigma_i = 1, \sigma_j = 1) = \int_0^{\Delta t}\int_0^{\Delta t} 1_{t<t'} P(\sigma_i(t))P(\sigma_j(t')|\sigma_i(t))dtdt' + \int_0^{\Delta t}\int_0^{\Delta t} 1_{t'<t} P(\sigma_j(t'))P(\sigma_i(t)|\sigma_j(t'))dtdt' \quad \text{(S6)}$$

$$= \int_0^{\Delta t} \lambda_i dt \int_t^{\Delta t} CFP_{ij}(t')dt' + \int_0^{\Delta t} \lambda_j dt \int_t^{\Delta t} CFP_{ji}(t')dt'. \quad \text{(S7)}$$

The expression for $CFP_{ij}(t)$ gives

$$P(\sigma_i = 1, \sigma_j = 1) = \frac{1}{2}\lambda_i \left(o_{ij} + M_{ij}\frac{w_{ij}^2}{w_{ij}^2 + T_{ij}^2} \right) \Delta t^2 + \frac{1}{2}\lambda_j \left(o_{ji} + M_{ji}\frac{w_{ji}^2}{w_{ji}^2 + T_{ji}^2} \right) \Delta t^2 + O(\Delta t^3), \quad \text{(S8)}$$

Here $M_{i,j}$, $T_{i,j}$, $o_{i,j}$ and $w_{i,j}$ are obtained from CFP. $M_{i,j}$ is interpreted as the strength of the connection, $T_{i,j}$ as the latency. $o_{i,j}$ represents uncorrelated background activity and $w_{i,j}$ accounts for the width of the peak. We have assumed that in the absence of neuron j firing, the probability of neuron i firing in a small time bin is

$$P(\sigma_i = 1) = \lambda_i \Delta t, \quad \text{(S9)}$$

where we have assumed that $(o_{ij} + M_{ij})\Delta t \ll 1 = \lambda_i \Delta t$. Here the firing rate λ is taken to be the mean firing rate over time, which includes the responses to other neurons in addition to neuron i's baseline firing rate. This same probability in the small coupling limit ($J_{ij} \ll \theta_i, \theta_j$) in the MaxEnt model is derived as follows. Let J be the average value of J_{ij}. First, we approximate the partition function in the

small coupling limit as

$$Z \approx \sum_{\sigma_i=0,1} e^{\left(\theta^\top \sigma\right)} \tag{S10}$$

$$= \prod_i (1+e^{\theta_i}) \tag{S11}$$

with

$$r = \sum_i \langle \sigma_i \rangle = \left(\sum_i \lambda_i \right) \Delta t, \tag{S12}$$

which yields

$$P(\sigma_i = 1) \approx \frac{e^{\theta_i}}{1+e^{\theta_i}} \tag{S13}$$

and

$$P(\sigma_i = 1, \sigma_j = 1) \approx \frac{e^{(\theta_i+\theta_j+J_{ij}+J_{ji})}}{(1+e^{\theta_i})(1+e^{\theta_j})}. \tag{S14}$$

Now, we match these two models to get

$$\lambda_i \Delta t = \frac{e^{\theta_i}}{1+e^{\theta_i}}, \tag{S15}$$

and

$$e^{J_{ij}+J_{ji}} = \frac{1}{\lambda_i \lambda_j} \left(\frac{1}{2}\lambda_i \left(o_{ij} + M_{ij} \frac{w_{ij}^2}{w_{ij}^2 + T_{ij}^2} \right) + \frac{1}{2}\lambda_j \left(o_{ji} + M_{ji} \frac{w_{ji}^2}{w_{ji}^2 + T_{ji}^2} \right) \right), \tag{S16}$$

which solves as

$$J_{ij} + J_{ji} = \frac{1}{2} \log \left(\frac{1}{2} \frac{1}{\lambda_i \lambda_j} \left(\frac{1}{2}\lambda_i \left(o_{ij} + M_{ij} \frac{w_{ij}^2}{w_{ij}^2 + T_{ij}^2} \right) + \frac{1}{2}\lambda_j \left(o_{ji} + M_{ji} \frac{w_{ji}^2}{w_{ji}^2 + T_{ji}^2} \right) \right) \right), \tag{S17}$$

ignoring corrections of $O(\Delta t)$. Note that we can only make a statement about $J_{ij} + J_{ji}$. When it is safe to assume that $T_{ij} \ll w_{ij}$,

$$J_{ij} + J_{ji} = \frac{1}{2} \log \left(\frac{1}{2} \left(\frac{o_{ij} + M_{ij}}{\lambda_j} + \frac{o_{ji} + M_{ji}}{\lambda_i} \right) \right) \tag{S18}$$

Hence, $J_{ij} + J_{ji}$ directly reflects M_{ij}, M_{ji}, normalized by various other parameters related to the firing patterns and shifted by a factor that depends on the mean J_{ij} and the mean firing rate. Note also that while θ_i has a strong dependence on Δt, J_{ij} does not depend on the size of time bins to first order in Δt.

If one does not assume that Δt is small, additional terms can be added, yielding corrections of the form

$$e^{J_{ij}+J_{ji}} = \frac{1}{\lambda_i \lambda_j} \Big(\frac{1}{2}\lambda_i \left(o_{ij} + M_{ij} \frac{w_{ij}^2}{w_{ij}^2 + T_{ij}^2} \right) + \frac{1}{2}\lambda_j \Big(o_{ji} + M_{ji} \frac{w_{ji}^2}{w_{ji}^2 + T_{ji}^2} \Big) + \frac{2}{3} \left(\frac{\lambda_i M_{ij} w_{ij}^2}{(T_{ij}^2 + w_{ij}^2)^2} + \frac{\lambda_j M_{ji} w_{ji}^2}{(T_{ji}^2 + w_{ji}^2)^2} \right) \Delta t \Big), \tag{S19}$$

ignoring corrections of $O(\Delta t^2)$. This introduces another culture-dependent shift in the zeroth-order relationship between estimated MaxEnt functional connectivities and true MaxEnt functional connectivities from Eq. S17.

The timescale Δt must be small enough so that $\frac{J_{ij}+J_{ji}}{\min(|\theta_i|,|\theta_j|)}$ is small and so that the additional correction term shown above is typically small.

Computational load for MaxEnt and CFP scaled with the number of active electrodes and with the total number of recorded spikes. We applied MaxEnt and CFP to 1h recordings of spontaneous activity using HP Elite Desk 800 G5, processor Intel(R) Core(TM) i7-9700 CPU @ 3.00GHz 3.00 GHz, RAM 64,0 GB, 64-bit operating system. Figure S2 shows that CFP computational load increased exponentially with increasing number of active electrodes or total number of spikes, while MaxEnt computational time scaled with the number of data points.

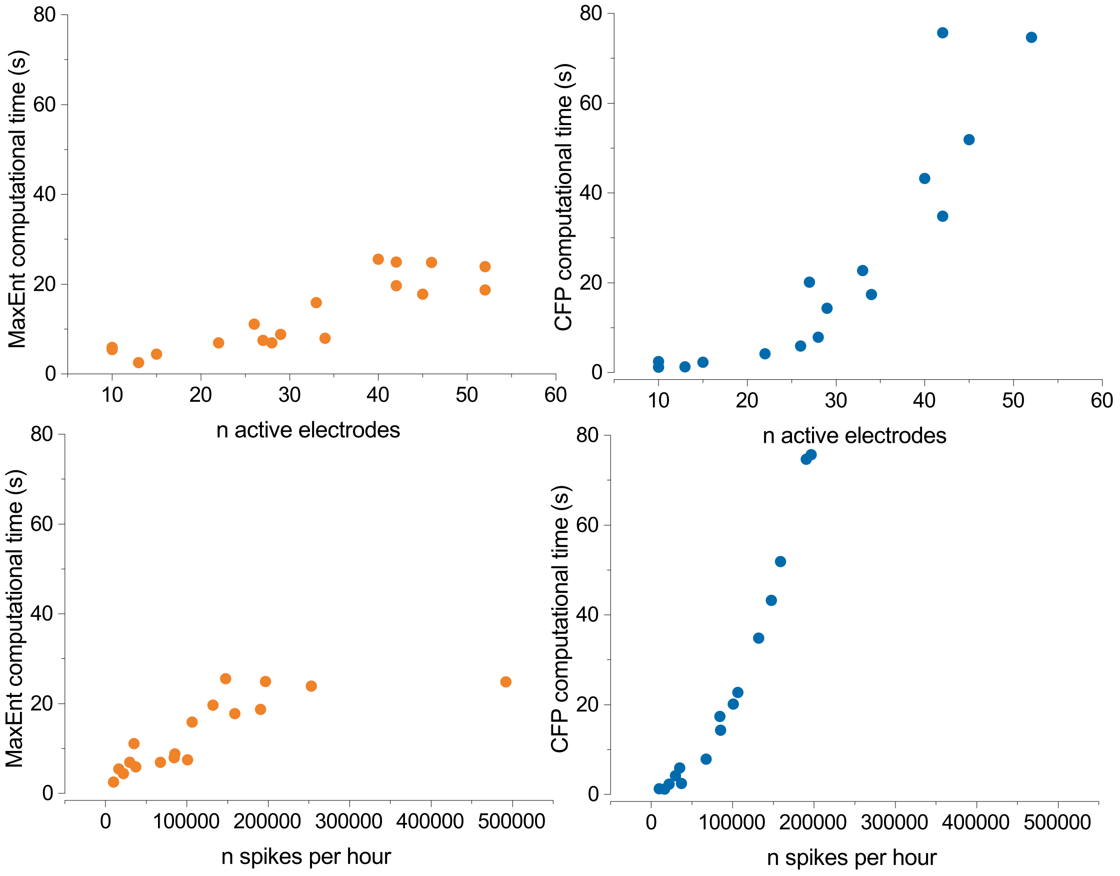

Figure S2: Relation between computational time for MaxEnt (orange) and CFP (blue) and data characteristics. Top graphs show relationship between computational time and the number of active electrodes (left: MaxEnt; right: CFP). Bottom graphs show computational time as a function of the number of spikes recorded in 1h (left: MaxEnt; right: CFP).

An analysis of the goodness of fit of Minimum Probability Flow

When finding parameters of our Maximum Entropy model, we are faced with the task of minimizing some sort of distance between model and data. In this case, we have

$$p_{model}(\vec{x}) = \frac{1}{Z} \exp\left(\vec{\theta}^{\top}\vec{x} + \vec{x}^{\top}J\vec{x}\right) \tag{S20}$$

where

$$Z = \sum_{\vec{x}} \exp\left(\vec{\theta}^{\top}\vec{x} + \vec{x}^{\top}J\vec{x}\right) \tag{S21}$$

is the partition function. Typically, practitioners take a maximum likelihood approach, in which we minimize the Kullback-Leibler divergence between model and data:

$$D_{KL}[p_{data}(\vec{x})||p_{model}(\vec{x})] = \sum_{\vec{x}} p_{data}(\vec{x}) \log \frac{p_{data}(\vec{x})}{p_{model}(\vec{x})} \tag{S22}$$

$$= \sum_{\vec{x}} p_{data}(\vec{x}) \log p_{data}(\vec{x}) - \sum_{\vec{x}} p_{data}(\vec{x}) \log p_{model}(\vec{x}) \tag{S23}$$

$$= -H[p_{data}] - \log L \tag{S24}$$

where $H[p_{data}]$ is the entropy of the data distribution and $\log L$ is the log likelihood. This well-known relationship between Kullback-Leibler divergence and log likelihood implies that minimizing an information-theoretic distance between the model and data distributions is equivalent to choosing parameters by

maximizing the (log) likelihood. Note that we have no control over the entropy of the data distribution, and so the $H[p_{data}]$ term is irrelevant to our parameter estimation procedure. If we now proceed further expand the log likelihood, we find

$$\log L = \sum_{\vec{x}} p_{data}(\vec{x}) \log p_{model}(\vec{x}) \tag{S25}$$

$$= \sum_{\vec{x}} p_{data}(\vec{x}) \left(\left(\vec{\theta}^{\top} \vec{x} + \vec{x}^{\top} J \vec{x} \right) - \log Z \right) \tag{S26}$$

$$= \vec{\theta}^{\top} \langle \vec{x} \rangle_{data} + \sum_{i,j} J_{i,j} \langle x_i x_j \rangle_{data} - \log Z \tag{S27}$$

where the $\langle \cdot \rangle_{data}$ indicate that an average is taken with respect to the data distribution. If we search for parameters J, θ that maximize the log likelihood, we look for parameters such that the gradient of the log likelihood is 0:

$$\nabla_{\theta} \log L = \langle \vec{x} \rangle_{data} - \langle \vec{x} \rangle_{model} \tag{S28}$$

$$\nabla_{J} \log L = \langle \vec{x}\vec{x}^{\top} \rangle_{data} - \langle \vec{x}\vec{x}^{\top} \rangle_{model}, \tag{S29}$$

where some straightforward manipulation has been omitted.

It seems that we have a straightforward procedure for finding J, θ, in which we try to maximize $\log L$ via some sort of gradient ascent using the gradients calculated above. But the averages with respect to the model distribution are fraught with difficulty, since we must (naively) sum over all possible $\vec{x}$. This can be prohibitively expensive. For example, suppose that there are 60 neurons– then there are 2^{60} possible binary vectors.

The typical way around this problem is to use contrastive divergence[2], in which some form of Monte Carlo is used to approximate the model averages $\langle \vec{x} \rangle_{model}$, $\langle \vec{x}\vec{x}^{\top} \rangle_{model}$. But about a decade ago, an alternative approach was discovered. Instead of trying to minimize the Kullback-Leibler divergence between data and model distributions, in Minimum Probability Flow (MPF), we imagine that there is some dynamics that takes the data distribution to the model distribution and try to minimize $D_{KL}[p_{data}||p_t]$, where p_t is the transformed data distribution a time t later. This gives the following objective function

$$K(\theta, J) = D_{KL}[p_{data}||p_t(\theta, J)] \tag{S30}$$

For small t this reduces to an objective function of

$$K(\theta, J) = D_{KL}[p_{data}||p_t(\theta, J)]|_{t=0} + t \left(\frac{\partial D_{KL}[p_{data}||p_t(\theta, J)]}{\partial t} \right) \Bigg|_{t=0} \tag{S31}$$

Then, following the derivation by Sohl-Dickstein and colleagues (Eqs. A1 - A9 of Appendix A)[3], we obtained

$$K(\theta, J) = \sum_{\vec{x} \in \mathcal{D}} \sum_{\vec{x}' \notin \mathcal{D}} g_{\vec{x}, \vec{x}'} \exp \left(\frac{E(\vec{x}') - E(\vec{x})}{2} \right) p_{data}(\vec{x}) \tag{S32}$$

where $\mathcal{D}$ is the set of binary vectors in the data, $E(\vec{x}) = \vec{x}^{\top}\theta + \vec{x}^{\top} J \vec{x}$ and g_{ij} is an arbitrary symmetric connectivity factor. This is the probability flow that we try to minimize. An appropriate choice of g_{ij} yields a highly tractable objective function, and regardless of the choice of g_{ij}, this objective function is convex. (We used $g_{ij} = 1$ only when i and j were one bit flip away.) It was shown in Refs.[3] that when p_{data} could be fit exactly by a Maximum Entropy model, MPF would find that Maximum Entropy model very quickly– more quickly than contrastive divergence, for example.

We first ask whether or not MPF will do a good job of fitting a data distribution that is likely not exactly a Maximum Entropy model. We simulated a small number of simulated neurons (small enough that contrastive divergence is not required) and suppose that $p_{data}(\vec{x})$ is chosen from a Dirichlet distribution. Then, there is no real structure to speak of in $p_{data}(\vec{x})$. It is still a well-posed question to find the best possible Maximum Entropy model. But now, MPF and maximum likelihood might yield different answers. In addition to log likelihood, we monitor the total variational distance $TVD = \sum_{\vec{x}} |p_{data}(\vec{x}) - p_{model}(\vec{x})|$ between the model and data distributions. We find that, with enough data, they yield almost indistinguishable answers in terms of goodness of fit. Fig. S3 shows an example run.

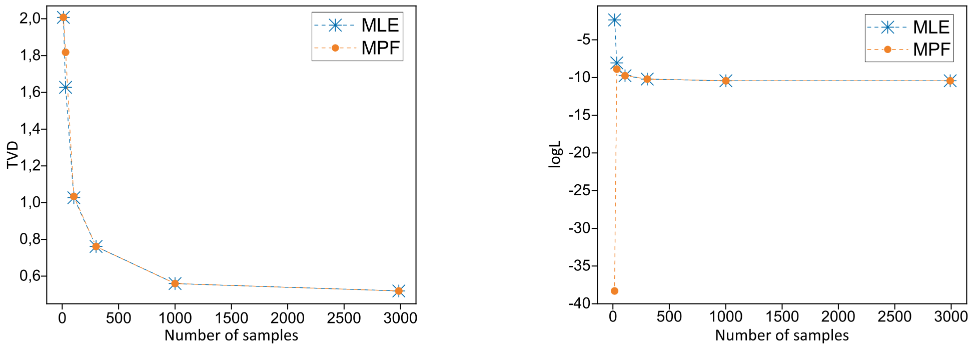

Figure S3: A distribution over binary vectors was randomly drawn from a Dirichlet distribution with concentration parameter 1 and then fit with both MPF and MLE using varying numbers of samples. At left, the total variational distance between model and data distributions. Increasing the number of samples causes MLE to better fit the model to the data, while it results in essentially no change for MPF. At right, the log likelihood of the data under the model when fit using MPF and MLE; high likelihood is better. When MPF data points are not present, the log likelihood is negative infinity.

However, closer inspection reveals that the model and data can match without the inferred connectivity matching the true connectivity well. We simulated a neural system that behaves according to a known MaxEnt model, and then inferred parameters J and θ using MPF and MLE. The ground truth J's and θ's did not match the inferred J's and θ's, even though the first-order, second-order, and third-order moments all matched. This is a typical property of a sloppy model[4]. This implies that we cannot completely trust the J's and θ's inferred by either MLE or MPF. It is therefore somewhat of a surprise that the results in the main text validate the match between CFP and inferred MaxEnt functional connectivities.

Finally, unexpectedly, MPF does not seem to be correctly fitting the MaxEnt model on the real neural data, in that the moments of the MPF-fitted MaxEnt model do not match the first and second-order moments of the experimental data. We checked this by using a Markov Chain Monte Carlo sampler (validated first on a subset of twenty neurons) on the 60-neuron MPF-fitted MaxEnt model and comparing the calculated first and second-order moments to the first and second-order moments of the experimental data. There was poor agreement as shown in Fig. S4. This could be a product of the sampler used, as proposal distribution can greatly affect results.

The last two facts combined lead us to a surprising conclusion. The MPF-inferred MaxEnt functional connectivities do not match the functional connectivities that we would infer if we demanded exact matches with the first and second-order moments. But the results in the main text suggest that the MPF-inferred MaxEnt functional connectivities are still correlated with the functional connectivities that we would infer if perfect matches with first and second-order moments were demanded. We can make no guarantees about functional connectivities inferred using contrastive divergence or other methods.

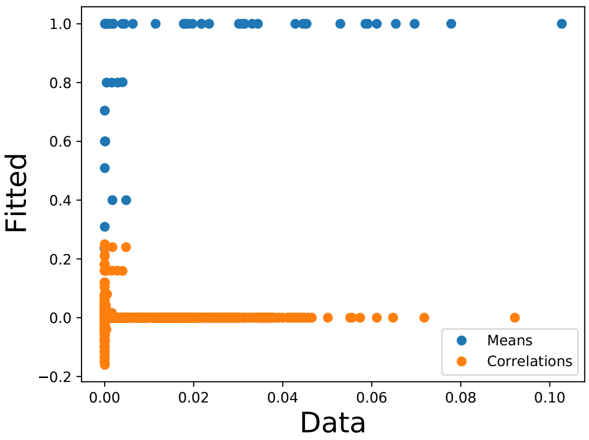

Figure S4: A scatterplot between the predicted means $\langle x_i \rangle$ and correlations $\langle x_i x_j \rangle - \langle x_i \rangle \langle x_j \rangle$ and the means and correlations from the data for one representative experiment. The data show a wide range of means and correlations, but the predicted means and correlations tend to be 1 and 0, respectively.

References

[1] Cocco, S., Leibler, S. & Monasson, R. Neuronal couplings between retinal ganglion cells inferred by efficient inverse statistical physics methods. *Proceedings of the National Academy of Sciences* **106**, 14058–14062 (2009).

[2] Hinton, G. E. Training products of experts by minimizing contrastive divergence. *Neural computation* **14**, 1771–1800 (2002).

[3] Sohl-Dickstein, J., Battaglino, P. B. & DeWeese, M. R. New method for parameter estimation in probabilistic models: minimum probability flow. *Physical review letters* **107**, 220601 (2011).

[4] Machta, B. B., Chachra, R., Transtrum, M. K. & Sethna, J. P. Parameter space compression underlies emergent theories and predictive models. *Science* **342**, 604–607 (2013).
